# Clinical outcome measures reporting in randomized trials evaluating Tuina therapy for chronic nonspecific low back pain: A systematic review

**DOI:** 10.1097/MD.0000000000033628

**Published:** 2023-04-21

**Authors:** Xuan Zhou, Qingyu Ma, Juan Yang, Arya B. Mohabbat, Ivana T. Croghan, Celia la choo Tan, Jiaxu Chen, Brent A. Bauer

**Affiliations:** a Formula-Pattern Research Center, School of Traditional Chinese Medicine, Jinan University, Guangzhou, China; b Division of General Internal Medicine, Mayo Clinic, Rochester, MN; c Department of Physiotherapy, Singapore General Hospital, Singapore.

**Keywords:** Chinese medicine, low back pain, massage, measurement, outcome, Tuina

## Abstract

Tuina has gained widespread attention and utilization for the management of chronic nonspecific low back pain (CNLBP). However, evidence-based guidance for choosing accurate and appropriate outcome measures of Tuina is lacking. The objective of this study is to systematically synthesize the existing outcome measures reported in randomized controlled trials (RCTs) evaluating Tuina therapy in CNLBP.

**Methods:**

Electronic literature searches were conducted in multiple English and Chinese databases from their inception to May 2022. RCTs were included if they involved clinical outcome measures in Tuina treatment for patients with CNLBP. Outcome instruments for each study were extracted and analyzed. Evidence from included studies were assessed using the Cochrane risk-of-bias tool.

**Results:**

Of the 735 identified articles, 17 articles with 1628 participants were included. Measurement domains in these RCTs were mainly reported in terms of pain (94%) and physical activity (71%), followed by safety (41%), Chinese medicine outcome (35%), and quality of life (12%). Moreover, several limitations with existing outcomes were reported, including lack of emphasis on the evaluation of quality of life, inadequate safety monitoring, as well as insufficient and vague Chinese medicine outcome measures. All trials were deemed to be of poor methodological quality.

**Conclusion:**

Pain and physical disability were the most frequently studied outcome domains in CNLBP treated by Tuina therapy. More rigorous and high-quality trials with appropriately selected outcome measures are needed in the future.

## 1. Introduction

Low back pain is a major public health concern and is associated with approximately 57.6 million person-years of disability globally.^[1]^ About 90% of low back pain cases are without a specific underlying cause; these cases are categorized as nonspecific low back pain.^[2,3]^ When nonspecific low back pain lasts for more than 3 months, it is termed chronic nonspecific low back pain (CNLBP).^[4]^

Treatment goals in CNLBP are to reduce pain, restore function, and prevent recurrence.^[4]^ Currently, the mainstays of treatment for CNLBP consist of conservative nonpharmacological options, minimally invasive procedures, and functional rehabilitation.^[5,6]^ In terms of nonpharmacological interventions, Chinese medicine interventions such as acupuncture, massage, herbs, and cupping etc. have increased in popularity, providing a unique therapeutic framework for pain relief and physical function improvement.^[4]^

Tuina, also referred to as Chinese massage, is a traditional nonpharmacological treatment option that utilizes manual manipulation of specific body parts to improve various conditions.^[7]^ It has been widely recommended to patients with low back pain throughout East Asian countries.^[8]^ Although it has been frequently utilized to treat CNLBP, there is a paucity of efficacy evidence, and therefore Tuina has not been widely recognized as a potential therapeutic option.^[9]^ Numerous clinical trials have been conducted and published on Tuina for CNLBP.^[10–26]^ Despite these trials, the summative evidence for Tuina in CNLBP remains unclear; this is due in part to the utilization of different Tuina techniques, heterogeneity of conditions studied, and differing protocols. All these challenges are further exacerbated by the disparity of outcome measures used in existing studies.

In this review, we aim to systematically appraise the outcome measures of identified randomized controlled trials (RCTs) assessing the therapeutic effect of Tuina therapy for CNLBP. By doing so, we aim to objectively study the clinical utility of Tuina and address the existing literature gap.

## 2. Methods

### 2.1. Literature search strategy

Comprehensive literature searches were performed in 3 English databases (OVID, SCOPUS, and Clinic Trials) and 4 Chinese databases (Chinese Biomedical Literature Database, Chongqing VIP Chinese Scientific Journals database, China National Knowledge Infrastructure, and Wanfang database) from their inception to May 2022 in both English and Chinese languages. MeSH terms and key words included low back pain, chronic low back pain, chronic zygapophyseal joint pain, chronic discogenic low back pain, chronic lumbar muscle strain, and chronic sacroiliac joint pain. Treatment interventions included (but not limited to) Tuina, Tui-na, Tui Na, massage, and Chinese Massage. No limitation to comparators or outcomes were included to avoid missing any relevant RCTs. Two authors independently assessed the eligibility of each record; any dispute was resolved by a discussion between the 2 authors or a consult with a third author.

### 2.2. Ethics approval

This study was a systematic review of published studies, no ethics approval was required.

### 2.3. Study selection and data extraction

#### 2.3.1. Inclusion and exclusion criteria.

Any RCT with participants diagnosed with CNLBP, based on the European guideline Classification of CNLBP criteria,^[27,28]^ were included. There was no restriction on age, gender, race, ethnicity, or duration of disease. Any technique of manual Tuina therapy was included. Control interventions included usual care, sham Tuina/placebo, or no treatment/wait list control.

Exclusion criteria were trials that were non-RCT design, non-CNLBP diagnosis, duplicated studies, cellular studies, direct comparison of different types of Tuina, animal experiments, or studies with missing data.

#### 2.3.2. Data extraction and quality assessment.

The following data points were collected by 2 authors independently from each study: study characteristics, eligibility criteria, interventions, outcome measurements, and safety information. Any disagreement was resolved via discussion or consultation with a third author.

The methodological quality of the included publications was assessed by 2 independent authors, using a collaboration tool recommended by the Cochrane Handbook.^[29]^ Seven domains were evaluated: random sequence generation, allocation concealment, blinding of participants, personnel, outcome assessors, incomplete outcome data, selective outcome reporting, and other sources of bias.^[30]^ Each domain in the assessment was judged as potentially having a low, unclear, or high risk of bias. Any inconsistency between the 2 reviewers was resolved through consensus or additional consultation with a third author.

#### 2.3.3. Data analysis.

Due to the qualitative differences between different measures within each outcome domain, a meta-analysis was not performed. Descriptive statistics for the outcome measures reported in the search were provided for each domain and a summary of methods was presented to develop a core outcome set.

## 3. Results

### 3.1. Trial identification

**Figure 1.**
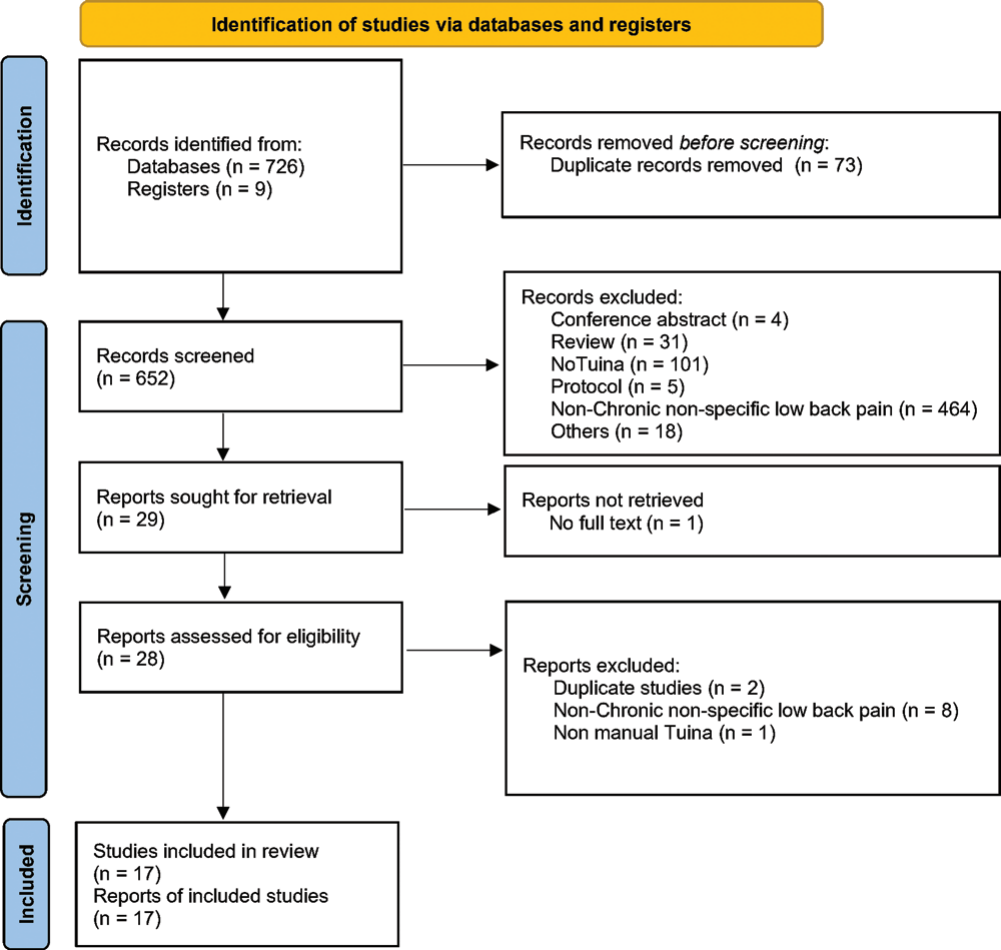
Flow chart of included studies.

The initial electronic literature search identified 735 relevant records. After removing 73 duplicate records, 652 publications remained. After a thorough analysis, a total of 17 published trials were identified and included in the review. The PRISMA flow diagram of the study selection process is shown in Figure 1.

### 3.2. Characteristics of included studies

A total of 17 studies involving 1628 participants met the eligibility criteria. The age of the study participants ranged from 18 to 75 years. All included trials were published between 2005 and 2021. All studies were conducted in China. Except for three three-arm studies,^[14,16,24]^ all other studies are 2-arm trials.^[10–13,15,17–23,25,26]^ The observed interventions varied among the 17 studies, including Tuina alone,^[13,15,19,20,22–25]^ Tuina plus physiotherapy,^[10,17]^ and Tuina plus the other Chinese medicine options.^[11,12,14,16,18,21,26]^ Characteristics of the included studies are summarized in Table 1.

**Table 1:** Characteristics of included studies (n = 17).

Author	Year	Arm	N	Age	Tuina relevant intervention	Pain	Physical disability	QoL	TCM	Safety	Other
Han et al	2005	2	122	22–49	Tuina	X	Self-made low-back lumbar spine function score	X	X	X	X
Zheng et al	2012	2	64	21–70	Lumbar tender point deep tissue massage plus lumbar traction	VAS, pressure pain threshold and muscle hardness of the tender point	X	X	X	X	X
Yuan et al	2014	2	110	28–60	Tuina	VAS	ODI	X	X	AEs	X
Sun et al	2015	2	120	33–47	Sling exercise therapy and Tuina	VAS	ODI	X	X	X	X
Song et al	2015	2	160	23–56	Lijincutong techniques combined with movable cupping on the bladder meridian therapy	VAS	X	X	Self-made Low back pain symptom score	AEs	X
Feng	2016	3	90	36–59	Sling and Plucking Corresponding Meridian	VAS	JOA, Back extensor muscle strength and the flexibility of lower limb	X	x	X	X
Wang et al	2017	3	96	42–48	Shen’s Mang-needle combined Tuina on belt vessel	VAS	X	X	X	X	X
Liu	2018	2	60	25–75	Dredging meridian and regulating viscera Tuina therapy	VAS	X	X	Self-made Chinese medicine symptom score	X	sEMG
Jia	2018	2	68	39–63	Amelioration therapy of Gongting Therapeutic for Injured Soft Tissue	NRS	ODI, self-made physical disability measurement scale	X	X	AEs	X
Chen and Jiang	2018	2	120	23–57	Sinewing tendon therapy plus cupping on Bladder meridian	VAS	X	X	Self-made Low back pain symptom score	AEs	IL-6, TNF-α
Zhang	2019	2	86	44–78	Musk pain relief plaster + Tuina therapy of Dredging meridian and regulating viscera	VAS	ODI	X	Self-made Chinese medicine symptom score	X	X
Jiang et al	2019	2	128	25–70	Dredging meridian and regulating viscera therapy + Cheezheng pain relieving plaster	VAS	X	SF-36	Therapeutic efficacy evaluation	X	X
Yu	2019	3	81	20–48	Exercise intervention + acupuncture and Tuina	VAS	ODI, Core muscle endurance and explosive strength test	X	X	AEs, vital signs	X
Yao et al	2020	2	65	24–40	Sinew-regulating bone-setting manipulations plus exercise therapy	VAS	ODI, Detection of lumbar muscle endurance	X	X	AEs	sEMG
Feng	2020	2	60	18–58	Channels-acupoints dredge plus pain point knead dial Tuina manipulation combined with core stability training	VAS	RMDQ	SF-36	X	AEs	β-EP, IL-6, TNF-α
Lin	2020	2	120	28–65	Therapeutic manipulation for tendon injury of Tuina	Self-made chronic nonspecific low back pain symptom and sign scale,	JOA Scale	X	X	X	X
Zhao	2021	2	78	28–71	Tuina and Warm needle	VAS	ODI	X	Self-made Chinese medicine syndrome score	X	X

β-EP = beta-endorphin, AEs = adverse events, JOA = Japanese Orthopedic Association, IL-6 = interleukin-6, NRS = numeric rating scale, ODI = Oswestry disability index, RMDQ = Roland Morris Disability Questionnaire, sEMG = surface electromyography, SF-36 = short form-36, VAS = Visual Analogue Scale.

### 3.3. Risk of bias within studies

**Figure 2.**
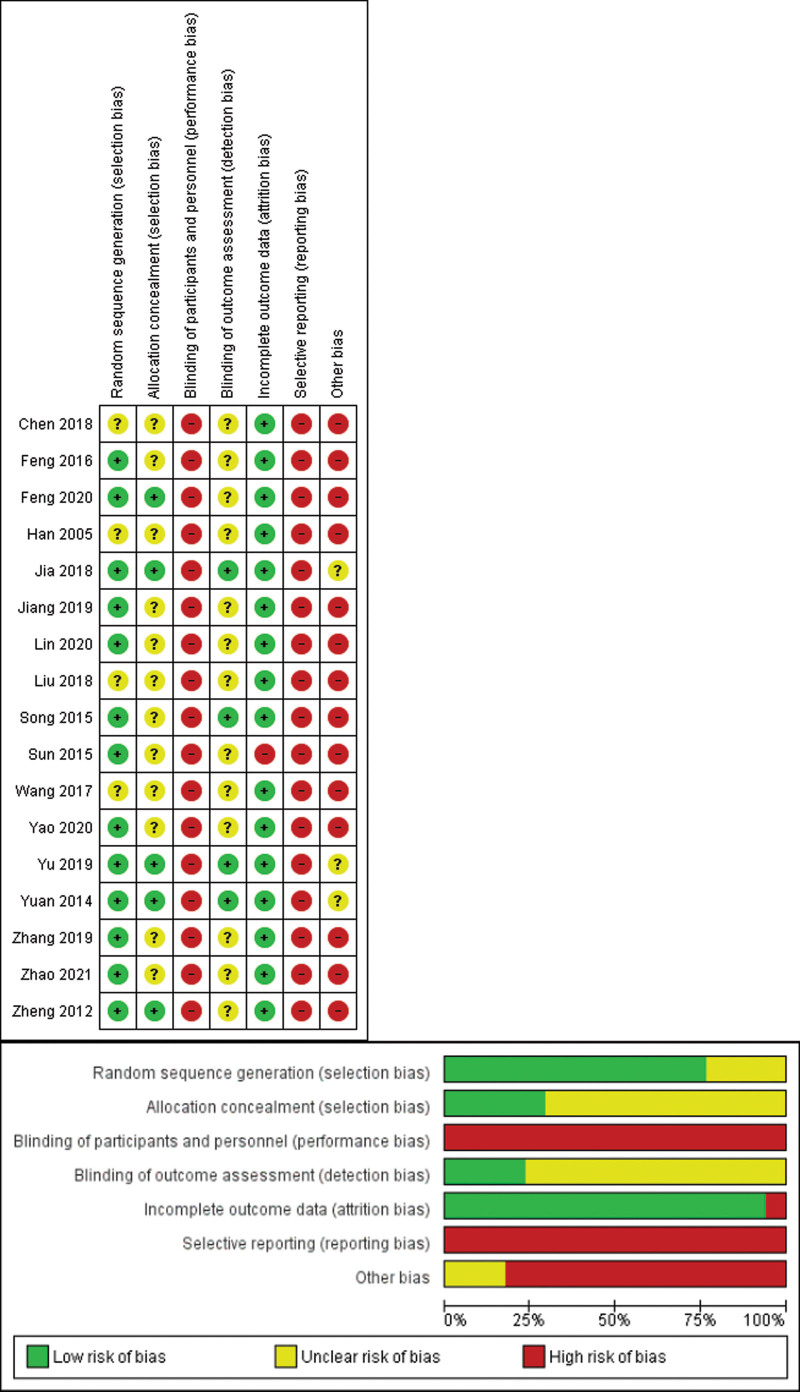
Risk of bias within included studies.

Among the 17 RCTs, only 13 used a “random number table,”^[11,12,14,15,17,18,20–22,24,25]^ “Microsoft Office Excel,”^[10]^ or “SAS software”^[13]^ to generate a set of random numbers; the others simply mentioned “random” without describing the specific methods.^[16,19,23,26]^ Five studies described allocation concealment with “sealed opaque envelopes,”^[10,13,14,22,25]^ while the other 12 did not give detailed information.^[11,12,15–21,23,24,26]^ All studies were considered to have a “high risk” of bias due to the absence of both participant and personnel blinding. Four studies were assessed as “low risk” in terms of blinding of outcome assessment,^[13,14,18,22]^ while the others were evaluated as “high risk” because there was no detailed description of how missing items were handled.^[10–12,15–17,19–21,23–26]^ All included trials were deemed as “high risk” in terms of reporting bias given the lack of trial outcomes in the protocol or registration data. Three trials had an “unclear risk” of other bias,^[13,14,22]^ while the other 13 had “high risk” of other bias.^[10–12,15–21,23–26]^ In general, the risk of bias of the included RCTs was assessed as “high” in Figure 2.

### 3.4. Outcomes reporting in clinical trials of Tuina for CNLBP

Diverse outcome measures were reported in the included RCTs, which were categorized into 6 main domains consisting of 22 outcome measurements: pain, physical disability, quality of life (QoL), Chinese medicine outcomes, safety, and others. Except for 8 studies that utilized self-made tools,^[12,18–23,26]^ the other 7 utilized evaluation tools from previous trials only.^[10,13,14,16,17,24,25]^

Pain severity was the most frequently reported outcome measured, which was present in 16 (94%) of the included studies.^[10–22,24–26]^ Twelve studies measured pain via the Visual Analogue Scale alone^[11–19,21,24–26]^ and 1 trial utilized the Visual Analogue Scale and pressure pain threshold and muscle hardness.^[10]^ One trial utilized the numeric rating scale to assess changes in pain.^[22]^ And the remaining one applied self-made chronic nonspecific LBP symptom and sign scale to evaluate the pain variable.^[20]^

Physical disability outcomes were reported in 12 studies (71%), using 8 instruments.^[11–15,17,20,22–25]^ Oswestry disability index was the most frequently used physical disability outcome, present in 6 trials.^[12–15,17,22]^ Japanese Orthopedic Association Scale,^[20,24]^ Roland-Morris Disability Questionnaire,^[25]^ and lumbar spine function measurements^[14,15,22–24]^ were also utilized to measure physical function.

Seven of the 17 included studies (41%) included safety information^[13–15,18,22,25,26]^; adverse events were the most common outcome measurement tool,^[13–15,18,22,25,26]^ while vital signs were monitored in 1 study.^[14]^

Three instruments were used in 6 trials (35%) regarding Chinese medicine outcome domains.^[11,12,18,19,21,26]^ Self-made low back pain symptom score was reported in 2 trials, one^[18]^ was self-made based on the *Guidelines for Clinical Research on New Chinese Medicines-soft tissue* injuries,^[31]^ combined with low back pain symptom score part of the Japanese Orthopedic Association Scale, and the other^[26]^ was on *Guidelines for Clinical Research on New Chinese Medicines-soft tissue injuries*^[31]^ only. Chinese medicine therapeutic efficacy evaluation was reported in 1 trial^[21]^ referring to *Traditional Chinese Medicine (TCM) diagnosis and curative effect standards*.^[32]^ Self-made Chinese medicine symptom score was reported in the other 3 trials, one^[12]^ was developed on the *Guidelines for Clinical Research on New Chinese Medicines*,^[33]^ and the other^[11,19]^ was on *TCM diagnosis and curative effect standards* from 3 different references.^[32]^

QoL was evaluated in 2 studies (12%) using the Study 36-Item Short Form Health Survey.^[21,25]^

In addition to the above domains, lumbar surface electromyography,^[15,19]^ and inflammatory markers^[25,26]^ were also recorded.

## 4. Discussion

This study synthesized outcome measures from RCTs that assessed Tuina in CNLBP. A thorough literature search identified 17 articles, which were included in our systematic review. Pain intensity and physical disability were the most frequently studied outcome domains, followed by Chinese medicine outcomes, safety monitoring, and QoL. Overall, this systematic review noted a poor methodological quality of the included RCTs according to Cochrane Collaboration risk of bias analysis. Randomization allocation, blinding methods, and selective reporting were not fully performed in most studies, which significantly influenced the quality of evidence.

Chronic pain is a complex and multifactorial process, which can negatively impact patients’ QoL. CNLBP is one of the most prevalent chronic pain disorders with a significant impact on QoL and daily function.^[34]^ It is interesting to note that QoL outcome measures were not commonly utilized. According to the current data of the included trials, QoL was only reported in 12% of studies. This makes it difficult to draw a firm conclusion about the therapeutic impact of Tuina in terms of QoL. More studies assessing Tuina in CNLBP are needed to draw firmer conclusions.

Safety outcomes are important to help healthcare professionals identify harms and risks of interventions. The safety profile of Tuina remains controversial. In the present review, less than half of the articles reported safety monitoring; most simply reported adverse events. Therefore, it is strongly recommended to clinically monitor for the presence of any safety issues with the usage of Tuina therapy.

Chinese medicine outcome is one of the key domains evaluating Tuina therapy for CNLBP. Through accumulated practical experience over 2000 years, Chinese medicine has developed into a set of unique principles that are different from those of conventional medicine.^[35]^ Appropriate outcome measures in Chinese medicine can better reflect the TCM characteristics and improve treatment understanding and usage of various TCM methods. While various Chinese medicine outcome measures were reported in this review, all outcome tools were vaguely described and significantly varied from study to study. Furthermore, all Chinese medicine outcome instruments were defined without formal reliability or validity information. We proposed that Chinese medicine outcomes should focus on the appropriate assessment of Chinese medicine syndrome when evaluating Tuina therapy in treating CNLBP. High quality, multicentre studies, to further assess the validity and reliability of these Chinese medicine outcome scores across various demographics, could generate more convincing evidence.

Given the current state of variability in study design and outcome measures in the existing literature, it is imperative to develop a core set of outcome measures for future clinical trials or clinical practice for all types of Chinese medicine interventions (including Tuina). For future Tuina trials and clinical utilization, we recommend a set of core outcomes to include pain, physical disability, QoL, safety, and TCM syndrome scale. This would help to overcome a variety of methodological barriers and provide robust evidence for Tuina therapy in CNLBP.

### 4.1. Strengths and limitations

This is the first study to systemically review outcome measures for Tuina in patients with CNLBP. This review synthesized data from both Chinese and English databases and thus produced a robust body of evidence. Across domains of outcomes, we identified an overall large treatment effect of Tuina in CNLBP. Furthermore, this review proposed a core set of outcome measures for future research and clinical practice.

There are several limitations that should be considered. Firstly, despite a thorough search strategy, we only included studies published in either English or Chinese languages. It is possible that additional studies in other languages could have been missed. Secondly, all included RCTs were conducted in China, which limits the generalizability, external validity, and strength of evidence. Thirdly, although RCTs are considered to provide the most reliable evidence for evaluating the effectiveness of interventions, the usage of a single study design could have failed to provide different insights into outcome measures. In addition, we did not include unpublished data and as such publication bias could not be avoided.

## 5. Conclusion

Tuina is a common treatment for CNLBP, however, optimal outcome measures in this clinical setting have not been clearly defined. Study findings indicated that pain and physical disability were the most frequently studied outcome domains. However, there were several concerns with the study design and chosen outcome measurements, including a lack of emphasis on the evaluation of QoL, inadequate safety evaluation, and the usage of insufficient and vague Chinese medicine outcome measures. As a result, the study findings should be cautiously interpreted due to the poor methodological quality of the included trials. Further rigorous and well-designed studies with a core set of outcome measures are needed for future studies for Tuina therapy in CNLBP.

## Acknowledgments

We would like to acknowledge the experienced librarian, Prokop, Larry J., Mayo Clinic Rochester Campus, for his significant contributions to the literature research and valuable, constructive advice.

## Author contributions

**Conceptualization:** Celia la choo Tan, Jiaxu Chen, Brent A. Bauer.

**Data curation:** Xuan Zhou, Qingyu Ma, Juan Yang.

**Formal analysis:** Xuan Zhou, Qingyu Ma, Juan Yang.

**Funding acquisition:** Celia la choo Tan, Jiaxu Chen, Brent A. Bauer.

**Investigation:** Brent A. Bauer.

**Methodology:** Ivana T. Croghan, Jiaxu Chen.

**Project administration:** Brent A. Bauer.

**Software:** Xuan Zhou, Juan Yang.

**Supervision:** Brent A. Bauer.

**Writing – original draft:** Juan Yang, Xuan Zhou.

**Writing – review & editing:** Celia la choo Tan, Jiaxu Chen, Brent A. Bauer, Arya B. Mohabbat, Ivana T. Croghan.

## References

[1] Vos T Abajobir AA Abate KH. Global, regional, and national incidence, prevalence, and years lived with disability for 328 diseases and injuries for 195 countries, 1990–2016: a systematic analysis for the Global Burden of Disease Study 2016. Lancet. 2017;390:1211–59. 28919117 10.1016/S0140-6736(17)32154-2 PMC5605509

[2] Zhu BY Li X Liao PD. [Academic features of tuina for children in western Hunan district]. Zhongguo Zhen Jiu. 2012;32:548–50. 22741266

[3] Maher C Underwood M Buchbinder R. Non-specific low back pain. Lancet. 2017;389:736–47. 27745712 10.1016/S0140-6736(16)30970-9

[4] Ma K Zhuang Z-G Wang L. The Chinese Association for the Study of Pain (CASP): consensus on the assessment and management of chronic nonspecific low back pain. Pain Res Manag. 2019;2019:1–14. 10.1155/2019/8957847 PMC6714323 31511784

[5] Chou R Deyo R Friedly J. Nonpharmacologic therapies for low back pain: a systematic review for an American College of Physicians Clinical Practice Guideline. Ann Intern Med. 2017;166:493–505. 28192793 10.7326/M16-2459

[6] Qaseem A Wilt TJ McLean RM.; Clinical Guidelines Committee of the American College of Physicians. Noninvasive treatments for acute, subacute, and chronic low back pain: a clinical practice guideline from the American College of Physicians. Ann Intern Med. 2017;166:514–30. 28192789 10.7326/M16-2367

[7] Jingwei L. Chinese massage and the introduction of massage into China before The 8th century. Anc Sci Life. 1986;6:24–9. 22557544 PMC3331398

[8] Cho H-W Hwang E-H Lim B. How current clinical practice guidelines for low back pain reflect traditional medicine in East Asian countries: a systematic review of clinical practice guidelines and systematic reviews. PLoS One. 2014;9:e88027. 24505363 10.1371/journal.pone.0088027 PMC3914865

[9] Yuan QL Guo TM Liu L. Traditional Chinese medicine for neck pain and low back pain: a systematic review and meta-analysis. PLoS One. 2015;10:e0117146. 25710765 10.1371/journal.pone.0117146 PMC4339195

[10] Zheng Z Wang J Gao Q. Therapeutic evaluation of lumbar tender point deep massage for chronic non-specific low back pain. J Tradit Chin Med. 2012;32:534–7. 23427384 10.1016/s0254-6272(13)60066-7

[11] Zhao L. Clinical observation on warm needle combined with massage in the treatment of chronic lumbar muscle strain. Clinical observation on warm needle combined with massage in the treatment of chronic lumbar muscle strain. Guangming J Chin Med. 2021;36:2021551068.

[12] Zhang Z. Shexiang Jietong ointment combined with massage in the treatment of 43 cases of chronic lumbar muscle strain caused by cold-dampness and blood stasis. J Med Pharm Chin Minorities. 2019;28:130–2.

[13] Yuan W Wang J Lu H. A randomized controlled study of Tuina therapy for chronic lumbar muscle strain. Treatment of chronic lumbar muscle strain by Tuina: a randomized controlled trial. Shanghai J Tradit Chin Med. 2014;48:82–5.

[14] Yu D. Clinical Effect of Exercise Intervention Combined with Acupuncture and Massage on Chronic Nonspecific Low Back Pain [master’s thesis]. Chengdu Institute of Physical Education; 2019.

[15] Yao ML Chen ZH Zhang WD. Clinical observation of sinew-regulating bone-setting manipulations plus exercise therapy for chronic non-specific low back pain. Article. J Acupunct Tuina Sci. 2020;18:59–66.

[16] Wang Z Yang X Liu B. Study on the treatment of chronic non-specific low back pain with Shen’s elongated needle therapy combined with massage. J Hebei Tradit Chin Med Pharmacol. 2017;32:45–46,52.

[17] Sun D Zhan Q Sun D. Clinical observation of suspension exercise training combined with massage in the treatment of chronic nonspecific low back pain. Effects of suspension training therapy and massage on chronic nonspecific low back pain. J Yunnan Univ Tradit Chin Med. 2015;38:82–5.

[18] Song F Hu J Zhang H. Lijincutong techniques combined with movable cupping on the bladder meridian therapy for lumbar muscle strain: clinical analysis of 80 cases. Lijincutong techniques combined with movable cupping on the bladder meridian therapy for lumbar muscle strain: clinical analysis of 80 cases. Liaoning J Tradit Chin Med. 2015;42:2015714678.

[19] Liu M. Treatment of chronic lumbar muscle strain with massage manipulation for clearing meridians and regulating internal organs. Clinical study on the treatment of chronic lumbar muscle strain by means of tunnel. J Changchun Univ Tradit Chin Med. 2018;34:934–6.

[20] Lin L. A clinical study on 60 cases of chronic non-specific low back pain treated with massage and tendon manipulation. Clinical study on 60 cases of chronic nonspecific low back pain treated by therapeutic manipulation for tendon injury of massage. New Chin Med. 2020;52:145–7.

[21] Jiang W Zeng P Zhang Y. Curative effect observation of Qizheng relieving pain paste combined with meridian-removing and internal-regulating massage in the treatment of patients with chronic lumbar muscle strain. Heilongjiang Med J. 2019;32:605–7.

[22] Jia D. Clinical Study on the Treatment of Chronic Non-specific Low Back Pain with the Modified Manipulative Manipulation of the Palace Tendons [master’s thesis]. Beijing University of Chinese Medicine; 2018.

[23] Han H Chen L Li L. Clinical analysis of 62 cases of chronic non-specific low back pain treated by massage manipulation. Clinical analysis of manipulation therapy for chronic nonspecific low back pain. Chin J Convalescent Med. 2005;14:97–8.

[24] Feng Z. Study on the Clinical Curative Effect and Mechanism of the Method of Hanging and Plucking along the Meridian in the Treatment of Chronic Lumbar Muscle Strain [master’s thesis]. Shandong University of Traditional Chinese Medicine; 2016.

[25] Feng Y. A Clinical Study on the Treatment of Chronic Non-specific Low Back Pain with Acupoint Dredging Plus Pain Point Rubbing and Massage Manipulation Combined with core Stability Training [master’s thesis]. Hubei University of Traditional Chinese Medicine; 2020.

[26] Chen X Jiang M. Treatment of chronic lumbar muscle strain with manipulative manipulation and bladder meridian and cupping and its effect on visual analogue scale of pain. Shanxi Med J. 2018;47:2923–6.

[27] Balagué F Mannion AF Pellisé F. Non-specific low back pain. Lancet. 2012;379:482–91. 21982256 10.1016/S0140-6736(11)60610-7

[28] Airaksinen O Brox JI Cedraschi C. Chapter 4 European guidelines for the management of chronic nonspecific low back pain. Eur Spine J. 2006;15:s192–300. 16550448 10.1007/s00586-006-1072-1 PMC3454542

[29] Cochrane. Cochrane Handbook for Systematic Reviews of Interventions. 2022. Available at: https://training.cochrane.org/handbook [accessed date May 25, 2022].

[30] Higgins J Green S. Cochrane Handbook for Systematic Reviews of Interventions Version 5.1. 0. London: The Cochrane Collaboration; 2011.

[31] Zheng X. Guidelines for Clinical Research on New Chinese Medicines – Soft Tissue Injuries. Beijing: China Medical Science and Technology Press; 2002.

[32] National Administration of Traditional Chinese Medicine. Traditional Chinese Medicine Diagnosis and Curative Effect Standard [S]. 1994.

[33] Zheng X. Guidelines for Clinical Research on New Chinese Medicines. Beijing: China Medical Science and Technology Press; 2002.

[34] Járomi M Szilágyi B Velényi A. Assessment of health-related quality of life and patient’s knowledge in chronic non-specific low back pain. BMC Public Health. 2021;21(Suppl 1):1479. 10.1186/s12889-020-09506-7 PMC8063275 33892680

[35] Chan E Tan M Xin J. Interactions between traditional Chinese medicines and Western. Curr Opin Drug Discov Devel. 2010;13:50–65. 20047146

